# Lemierre's Syndrome Following Extraction of Wisdom Teeth

**DOI:** 10.7759/cureus.11061

**Published:** 2020-10-20

**Authors:** Aarati Keshary, Margaret Hagan

**Affiliations:** 1 Internal Medicine, University of Kansas School of Medicine, Wichita, USA; 2 Infectious Disease, University of Kansas School of Medicine, Wichita, USA

**Keywords:** lemierres syndrome, wisdom teeth extraction, thrombophlebitis, lemierre's, tooth extraction

## Abstract

Lemierre’s syndrome is a rare but potentially fatal disease caused by oropharyngeal infections. It is characterized by thrombosis of the internal jugular vein leading to the systemic circulation of septic emboli. The common pathogen identified is Fusobacterium necrophorum. Our case involved thrombosis of the superficial internal jugular vein that developed after wisdom teeth extraction, a rarely reported complication.

## Introduction

Lemierre’s syndrome is referred to as “the forgotten disease,” as it is rarely seen in the antibiotic era. It is a potentially fatal disease most commonly due to oropharyngeal infections leading to thrombophlebitis of the veins, particularly the internal jugular vein [1-4]. This syndrome was first reported by Andre Lemierre in 1936 [5]. It is a form of septicemia commonly caused by Fusobacterium necrophorum*, *leading to metastatic infection. The infection most commonly arises from tonsillitis and mastoiditis but rarely as an odontogenic infection after teeth extraction [1-4]. The high mortality rate of approximately 18% necessitates emergent imaging and the initiation of antibiotic treatment [1]. We report an atypical case of Lemierre’s syndrome involving the superficial internal jugular vein following a wisdom teeth extraction. 

## Case presentation

An 18-year-old male with no significant medical history presented with a three-day history of nausea and vomiting that began with left jaw pain and swelling. He had an uneventful extraction of his wisdom teeth 20 days prior. On physical exam, he was found to be afebrile, tachycardic, and with a low blood pressure of 98/57 mmHg. Oxygen saturation was 99% on room air. The patient had a swollen and erythematous left jaw, and the abdominal exam revealed diffuse lower abdominal and left upper quadrant pain. His blood reports showed leukocytosis of 30.0x10^3/μL with 35% bands, elevated creatinine of 2.64 mg/dL, and lactic acid of 2.3 mEq/L. The coagulation profile demonstrated an international normalized ratio (INR) of 1.5 and a partial thromboplastin time of 39.5 seconds. The remainder of the blood work was unremarkable. A non-contrast maxillofacial computed tomography (CT) scan demonstrated bilateral extraction of maxillary and mandibular molars and bilateral cervical lymphadenopathy. There was soft tissue gas near the left mandibular extraction site along with induration within the subcutaneous soft tissues. An initial chest X-ray was unremarkable. A non-contrast CT of the abdomen revealed splenomegaly and few small nodes in the mesentery, which are nonspecific. The patient was initiated on intravenous (IV) broad antibiotic coverage with vancomycin and piperacillin+tazobactam, fluid replacement, and was admitted for further workup and treatment.

**Figure 1:**
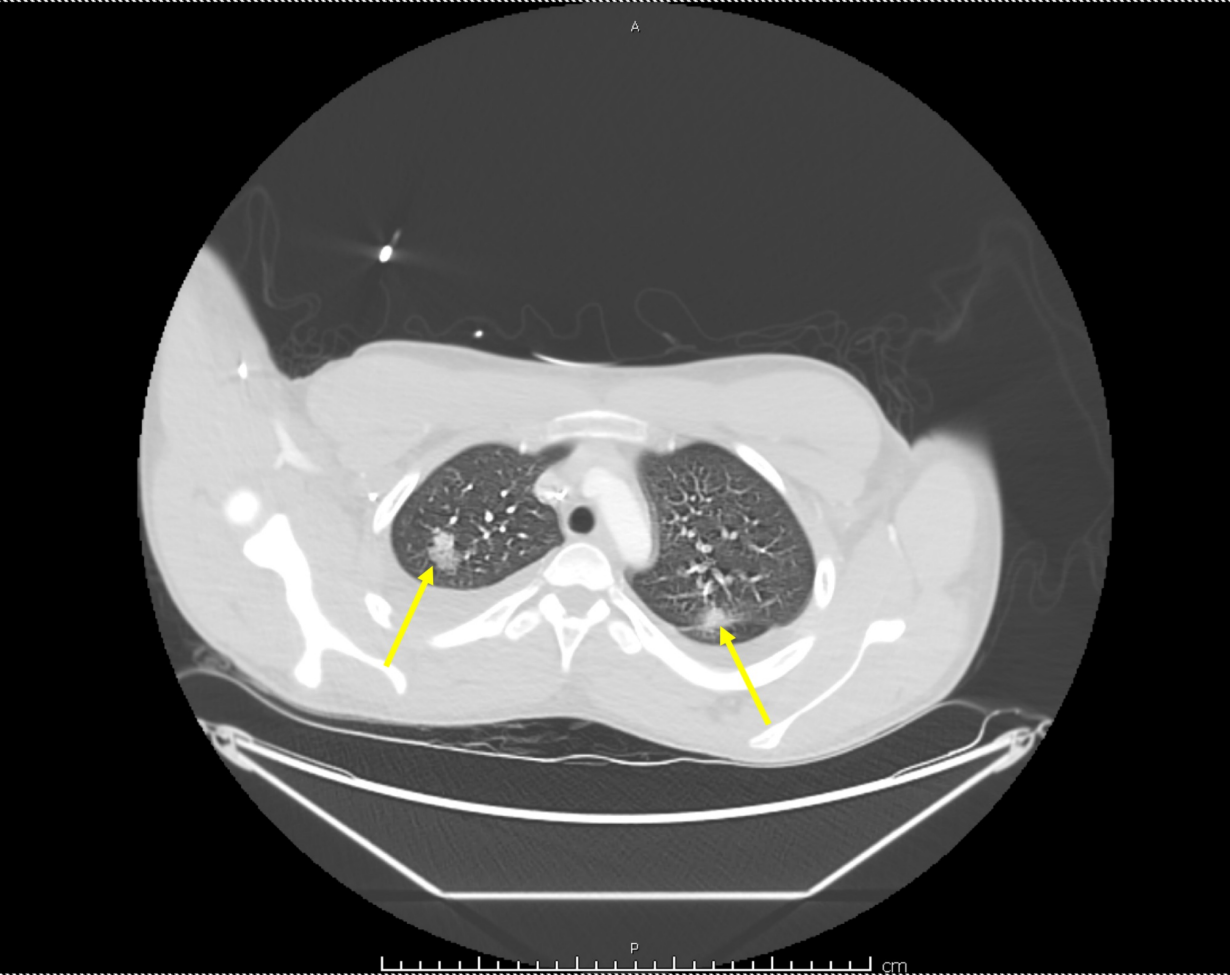
Chest computed tomography with contrast. It is illustrating bilateral pulmonary infiltrates (arrows).

**Figure 2:**
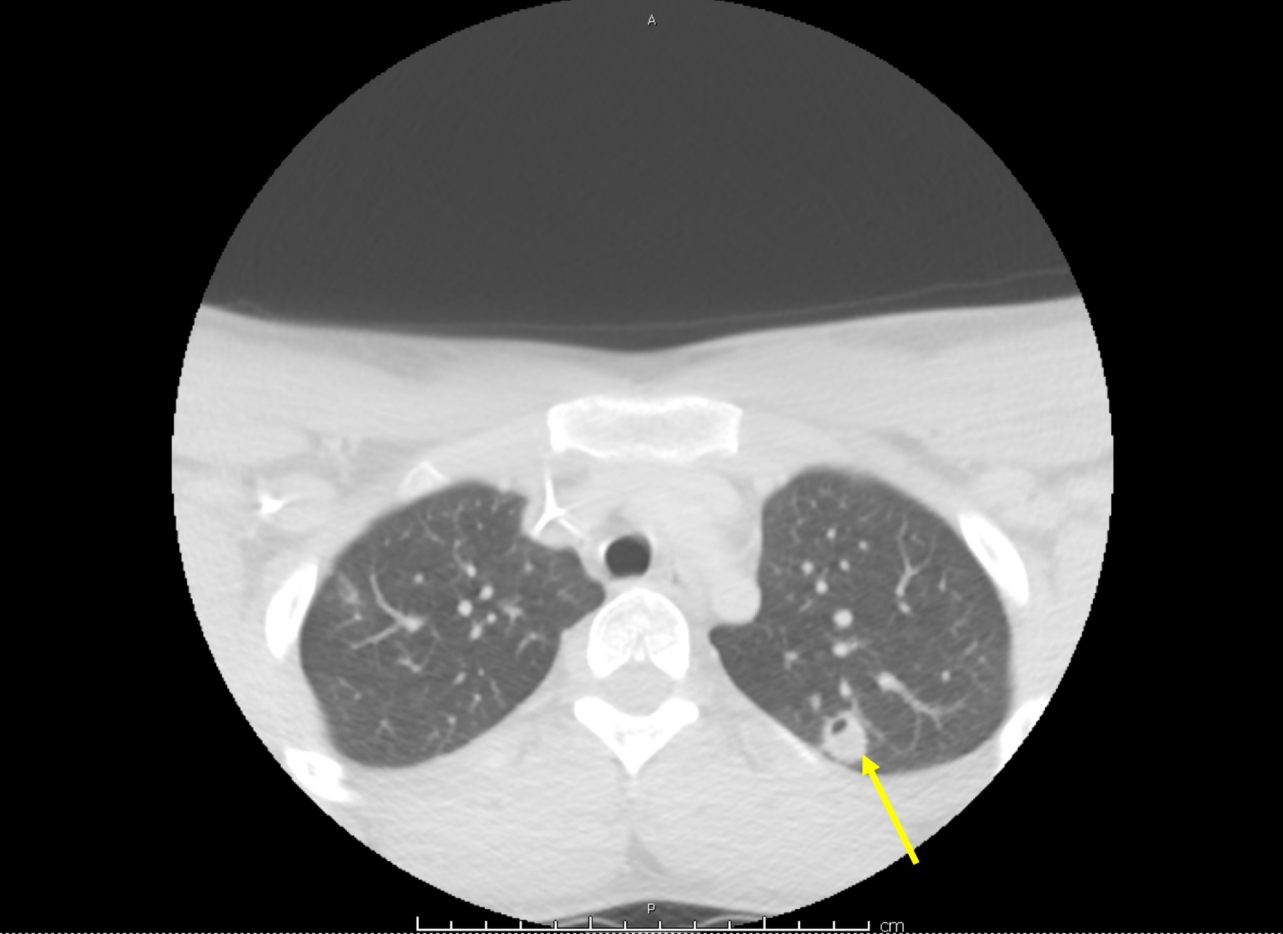
Chest computed tomography with contrast. Interval development of cavitation was seen later in nodular infiltrates consistent with septic emboli.

On day three of hospitalization, the patient developed severe acute hypoxic respiratory failure and was subsequently intubated. His condition deteriorated rapidly, leading to septic shock with multiorgan failure. An ultrasound of the neck showed a thrombosed superficial internal jugular vein consistent with thrombophlebitis. A repeat chest X-ray after intubation showed right lung opacities concerning for pneumonitis. CT of the head was unremarkable. CT angiography (CTA) showed scattered nodular-appearing infiltrates throughout both lungs and moderate bilateral effusion, as seen in Figure 1. An initial echocardiogram showed an ejection fraction of 30%. Blood culture from admission was positive forFusobacterium necrophorum. A follow-up echo showed an improvement of ejection fraction to 60%. Though no pulmonary emboli were identified on initial CTA, a subsequent CT scan showed evolving cavitation (Figure 2) of the numerous nodular densities seen on the initial chest X-ray consistent with septic pulmonary emboli. A vascular surgeon was consulted, who recommended no surgical intervention and to continue with medical management.

The patient improved clinically and was discharged on day 12. His follow-up CT of the jaw showed a periosteal reaction; therefore, he was treated with six weeks of ertapenem. One month after discharge, the follow-up radiographic imaging demonstrated resolution of the pulmonary infiltration, superficial internal jugular vein thrombosis, and improvement of the left mandibular osteomyelitis.

## Discussion

Lemierre's syndrome is caused by an oropharyngeal infection leading to thrombophlebitis of the veins, commonly the internal jugular vein [1-4]. Wisdom teeth extraction is a common routine medical procedure. There are very few reports of Lemierre’s syndrome following a wisdom teeth extraction [1]. The diagnosis is highly dependent on clinical presentation along with supporting laboratory results, including blood cultures and radiographic findings. CT scan with contrast can be used to confirm a thrombosed vein, most commonly the internal jugular vein, which is a deep vein. However, our case is atypical in that it presented with thrombosis of a superficial vein. The fact that the CT eventually revealed septic pulmonary emboli suggests that the deep venous system was involved. Broad-spectrum antibiotics are a key in the early treatment of sepsis, and in Lemierre’s disease, it is especially important to cover anaerobic organisms such as Fusobacterium [1-4]. There is no definitive treatment for thrombophlebitis; therefore, the use of anticoagulation is controversial and varies by case.

## Conclusions

Lemierre’s syndrome is referred to as the forgotten disease, as it is rare in the antibiotic era. The presence of odontogenic infection with worsening pain and neck swelling should raise high suspicion to obtain necessary blood cultures and imaging for early diagnosis. Given the high mortality rate, emergent imaging is crucial, and there should be no delay in the initiation of antibiotic treatment.
